# Counter-Irritation, Its Principles and Practice, Illustrated by One Hundred Cases, &c.

**Published:** 1838-10-01

**Authors:** 


### Counter-irritation, its Principles and Practice, illustrated by One Hundred Cases, &c. By *A.B. Granville*, M.D. &c. Octavo. Churchill, London. 1838. Pp. 360.

We are friends to counter-irritation, and daily witness its beneficial effects. But we are not quite so sanguine, as Dr. Granville seems to be, that the antidynous ammoniated lotion will go far to knock up the drug-trade, or, at all events, " save the constitution of patients from the pernicious effect of a polypharmacous treatment." We regretted to read the following passage in the preface. " I address it (the volume) in an especial manner to the general reader, rather than to my professional brethren." The reason for this is, that medical men will not be able to introduce the measure of counter-irritation widely, unless the public are first impressed with the importance of the remedy. " Of that importance the medical profession are fully aware: they have therefore little or nothing to learn from me on that point,"

We must pass over several sections on the terms which have been employed in designating the agency of counter-irritation—on the nature of counter-irritation—on the conditions necessary for its successful application—and on the various agents that have, from time to time, been employed—as the lytta, mustard, croton oil, &c. &c. In the sixth section the ammoniated counter-irritants—and particularly the " antidynous lotions," are brought forward. Dr. G. observes, that of 103 forms of counter-irritants, there is not one, of which we can " fix or determine the precise dose," that is necessary to produce the desired effect. This observation, however, applies pretty generally to all remedies. We can seldom ascertain the precise dose of calomel, jalap, or antimony, that will just do what we wish, and no more. Dr. Granville gives us no distinct formula for the ammoniated antidynous embrocation—unless the reader can extract it from the following passage.

"Some personal experiments made on myself, in the first instance, *with simple as well as compound preparations of ammonia, spirits of wine, vegeto-aromatic spirits, camphor, and other stimulating and evaporable substances, differing from the few preparations already in use, and combined with water, or with oils, or butyraceous vehicles, or saponified into cerates*—which experiments were afterwards repeated with the greatest success on some of the patients of two of the public medical institutions I belong to—led me to the knowledge of the fact, that, by merely regulating the several proportions of those ingredients, according to the nature and intensity of the case we have to treat; and, what is even more important, by mixing those proportioned ingredients in a particular order, instead of at random (paying due attention also to time), combinations of a more powerful kind than usual could be obtained. I found, in fact, that without charring the skin, or producing an eschar, such combinations would, on a mere application to the external surface of the body, give rise to peculiarly energetic effects on the disease, in the brief space of a very few minutes (sometimes seconds only), without necessarily producing at the same time rubefaction, vesication, and cauterization;—although, if sufficient time for the purpose were allowed, the same combinations would produce the latter phenomena also, besides the mere first impression."* 52.

In the 7th section are enumerated the occasions on which the antidyne (or anodyne, which is a better term after all, and more familiar) is to be employed. These are numerous enough, viz. in cases of spasmodic and painful complaints—muscular and tendinous affections—morbid affections of the vascular system—anomalous diseases, &c. &c.

The following is the mode of applying the anodyne.

"All that we have to do, is, first to impregnate with the colourless and transparent liquid, either a piece of linen folded six or seven times to the size of the part intended to be covered, or a piece of thick and coarse flannel; and secondly, to lay either of these on the spot, pressing with the hand at the same time, very steadily and firmly, the said linen or flannel, over which there should be placed a thick towel, doubled several times, so that not only the evaporation of the lotion may be impeded, but the hand employed in pressing the application to the part may not suffer damage from any evaporation or from contact with the liquid. In some parts of the body more convenient than others, the readiest and most effectual mode of pressing down the application, is by tying over it a towel or thick bandage; but to this mode there is the objection that we cannot under certain circumstances, inspect the part as quickly and as often as is required, so as to judge, from the effect of the application, when to stop, or how long to persevere in using it." 84.

This antidyne, contrary to what its name imports, produces, as might be expected, more or less pain, according to the degree of sensibility in the individuals.

"The first impression made is that of excessive coldness; presently, a pricking or tingling of the part supervenes, in all respects like that which we experience in a limb that has been 'asleep,' when the blood returns into it. This feeling, which at first occurs only in insulated points of the part acted upon by the lotion, soon becomes more general, until it seems to occupy the whole of the surface; and then a sense of heat in the part is substituted for the first impres-

* We have italicised a few lines containing all the information which the author chooses to grant respecting the actual combination.

sion of cold,—which heat and tingling increase gradually and simultaneously, until they seem to become one single sensation, approaching in its nature as near as possible to that experienced when we hold the hand close to a blazing coal fire for a minute, or rather resembling the painful feeling of a severe burn produced by scalding oil or melting sealingwax dropped on the fingers." 87.

We doubt whether any of our brethren will be able to compound the ingredients of this lotion, so as to produce the following effects:

"It now remains for me to assert, as a crowning of all this, that so soon as the pain of the lotion is felt, that instant the inward pain, for the removal of which it was applied, is suspended and at last vanishes. Indeed, during a paroxysm of tic-douloureux of the face, and while the patient is under the most agonizing suffering from that complaint, the immediate cessation of that suffering which follows closely upon the application of the lotion, and as soon as the counter-irritating pain is set up, borders on the miraculous." 88.

As we now live in the age of miracles, we hope the foregoing demi-miracle will often be realized both by Dr. Granville, and those who may be fortunate enough to discover the real composition of the antidynous lotion.

"If we watch the successive changes that take place in any part of the body during the application of the ammoniated lotions to it, we observe, as was before stated, a lively crimson blush of the skin in the course of the first two minutes, the cuticle remaining tight. In another instant or two more, however, the cuticle is seen corrugated here and there in exceedingly fine folds of a peculiarly white opacity. These folds occur generally in bundles, which are placed apart from each other, and leave a space between, where the cuticle is still stretched; until, in the course of two or three minutes more, it is seen to rise, sometimes in round dots like small bubbles, at other times in patches of every shape and dimension,—in all of which a pale yellowish thin fluid will be found, on close examination, to have collected. In a very few minutes more these patches and dots unite together or become confluent, and give rise, at last, to a general vesication of the skin in the part, resembling altogether that which is produced by an ordinary blister. By this time the quantity of the counter-irritant with which the compress was charged has, partly through absorption (?), partly through evaporation, become exhausted; and if any greater effect from the counter-irritant employed be wished for, the quantity must be renewed and re-applied to the same part, so as to establish in it a downright ulcerative process, in order that we may obtain, as a desirable result, a purulent discharge from the surface. It happens very often, that these three several stages or phenomena of local and artificial counter-irritation, are produced in half the time here mentioned, by the lotions in question; but I have also known, on a few occasions, the third phenomenon, or the stage of cauterization, not to have been produced under a shorter period of time than two or three hours.

From what has just been stated, the reader will perceive, that in its progress from rubefaction to ulceration, the lotion becomes a real blistering liquid; nor will they fail to remark also, that whereas a common blistering plaster requires some hours to produce even its first impression of tingling or of something like pain,—the counter-irritating ammoniated, or antidynous lotion, on the contrary, will raise a full blister in less than ten minutes." 89.

We now come to the narrative of one hundred cases, occupying more than 250 pages of letter-press. We think a much smaller number of cases would have been amply sufficient to illustrate not the principles of counter-irritation, for they are well known, but the peculiar agency of the antidyne. It will not be necessary for us to notice more than a few of these cases.

*Case* 1. This was Mr. Riley, a gentleman who had consulted many of the faculty in this metropolis, with little benefit. While serving in Van Dieman's Land some three and twenty years before Dr. G. was consulted, he was wounded by a spear above the right hip, towards the spine. He was soon afterwards attacked by neuralgia, and the following extract from his own statement to Dr. Armstrong, in 1830, will convey some idea of the nature of the assaults.

" My peculiar case appears to be one of almost exclusive distress, arising from such constant recurrence of pain in every part of my person, that I know not when or where I am free from suffering. My shoulders, the entire of each side, arms, back, hips, sciatic nerves, all parts of the thighs, knees, legs, ankles, and feet, are equally visited. I go to bed or rise apparently in my usual proportion of health and spirits, when, suddenly (and as generally in the night as in the day) I am attacked with pain, alternating in throbs, varying in degree of acuteness, and in duration from one to twenty-four hours, and more particularly under every and the most trifling changes in the state of the atmosphere. Sometimes I am only affected in one place, but not unfrequently in four, five, and more places at the same time, *and often with such extreme intensity that I cannot well describe my experience,* except that it then appears to resemble the agony of those afflicted with the malady termed *tic-douloureux!*" 105.

Mr. Riley had found by experience that the quickest way of dispersing the paroxysm was by enveloping the part in flannel bandages, drawing them as tight as possible. These sufferings were much mitigated (even according to the patient's own account,) by the antidyne, and he lived two or three years afterwards, in comparative health, dying at last of atrophy.

*Case* 2. (No. IV. in the book). The Countess of ———, had a singular affection of the chin, with severe nervous head-aches. The pain of the chin was somewhat periodic, and, at times, unbearable, and, in the intervals, the parts were benumbed. The ammoniated lotion was applied, and the result was an instantaneous removal of the pain, and return of the natural sensibility of the part. Vesication was produced.

Dr. Granville candidly mentions some cases of tic-douloureux where the antidyne failed. We do not wonder much at this, for, in too many cases the neuralgia depends on changes of structure which no medicine can cure, and few can even relieve.

*Case* 3. (X). Count de ——— suffered much from periodical pain of the supra-orbital nerve, coming on at eight o'clock in the morning, and lasting till noon. The London doctors having failed, Mr. Scott, of Bromley, was employed, and exhibited large doses of bark, without benefit. St. John Long was equally unsuccessful. Then came our author, who applied the antidyne to the brow, during a paroxysm. " The spasmodic contractions were almost immediately suspended," and, at the expiration of two minutes, the pain was gone. At the end of three minutes a blister was raised. The complaint did not return.

*Case* 4. (XIV.). A gentleman was subject to incessant pain at the pit of the stomach, sometimes increased by pressure—sometimes not. There were sometimes, when the pain was very severe, an attendant nausea, and also palpitations. The antidyne, very strong, was used with partial, but not complete relief.

*Case* 5. (XVIII). The Countess of ——, came under our author's care in March, 1835, for a severe spasmodic complaint of the back, of eight months' standing, and attributed at first to a chill. The following is a graphic, we had almost said scenic description of one of the paroxysms, which usually came on at eight o'clock in the evening.

"When I entered the chamber, in which were the Duchess of ———, the lady's mother, and a female attendant, the agony of the patient must have been excessive,—judging by her contortions, the agitation and cramping of her limbs, and the severe pain of which she complained. She was laid flat on a couch, and her spine examined, when it was found that the whole length of it seemed in motion,—representing, not unaptly, the annular movements of a snake. Even the iliac bones were drawn up and down, with a jerk and violence of motion, such as I had never seen before, and should have deemed almost impossible, had I not seen it. The pain, like the electric fluid, shot up along the back-bone into the occiput, and thence through the head into the globes of the eyes, which became painful, rolled violently in their sockets, and gave a dismal character to the face, itself greatly agitated. The patient could just mutter a few words in answer to my questions, from which I learned that pain was then pervading the shoulders and arms as well as the lower extremities; that the surface of the abdomen felt sore on the slightest pressure, and especially so in the position she was then placed; lastly, that she experienced a sense of suffocation in the chest. There came on, while I was present, a severe cramp in the calves of the legs, and the feet felt very cold to the touch. As to the pulse, I found it next to impossible to count it, and the movements of the heart were equally rapid and irregular. Such was the violence of the attack, that although I endeavoured to press with some degree of firmness upon the back-bone, so as to keep it down in its natural position,—one of the many convulsive throes or spasmodic leaps of that part occurred more than once, which, by bending the spine almost double, anteriorly, threw off, like an inferior weight, the pressure of my hands. The scene was truly heart-rending, and not to be described in words. I prescribed and sent for a moderately-strong ammoniated lotion,—considering this to be a case in which such a counter-irritant might be of great service.

The preparation came, and I instantly applied a thick compress of linen, three inches square, saturated with it, on a portion of the spinal column, above the place which had been cupped and blistered during previous treatments. The watch was held by the mother. In three seconds the moaning ceased; in five seconds more the patient heaved a deep sigh; before the first minute elapsed, she said that 'the pain was going,' and presently 'that it was gone.' She exclaimed at the same time that 'the application was a blessing; it smarted much, but was a pleasure to her.' Two or three more deep sighs followed, which, I concluded, announced the cessation of the paroxysm. On inquiry afterwards, I learned that in general the diurnal paroxysms, which always lasted three hours, terminated in a succession of deep sighs. Wishing to make the matter more certain, I applied the same compress, still rather wet, on another and a little higher spot, and on a third place still higher, after having supplied the compress with a small quantity of fresh lotion. Altogether the three applications lasted three and a half minutes, during which the whole extent of the surface of the back-bone became red and hot, but no immediate blister followed." 151.

No medicine was given, and although some relapses occurred, yet the repeated applications of the antidyne restored the Countess to health and to society. No practitioner of experience can doubt, for a moment, that this was hysteria, and nothing else. It is by no means improbable that a bucket of cold water poured over the Countess would have stopped the paroxysms, just as well as the antidyne. It appears that the lady afterwards had attacks, in Scotland, in the country, and in London, but Dr. G. was not consulted, and he knows not how the lady was treated.

Several cases of epilepsy, sympathetic and idiopathic, are detailed, in which counter-irritation was more or less useful. But epilepsy is such a capricious disease—ceasing for months, or even years, without medicine at all—and afterwards recurring with accumulated ferocity—that we place little reliance on apparent cures. We have no doubt, however, that the counter-irritation in question would prove auxiliary to other means. We prefer the antimonial to the ammoniated applications in these cases, well knowing the superiority of a purulent over a serous discharge in chronic diseases. It is in sudden, violent, painful, and spasmodic attacks that the ammoniated lotion bids fair to be extremely useful.

Instances of spasmodic asthma and hay-fever are adduced, and where much benefit resulted from the application of the antidyne. But we are unable to dedicate more space to the remainder of a hundred cases detailed in this volume. They will be greedily perused by the public at large, and Dr. Granville will be obliged to erect an ammonia manufacture for the preparation of the antidyne.

---

Since writing the above we have made several experiments, first on our own persons, and afterwards on others, and we have no hesitation in averring that we can enable any man in Her Majesty's dominions to procure and apply this extremely useful and most valuable counter-irritant. This antidyne is neither more nor less than the *strongest* liquor ammoniæ that can be procured. The liquor ammonia of the Pharmacopœia, will not do; but the potent kind may be procured from most chemists. The following mode of application we have found to be the most easy and the most effectual. Fill the lid of a turned wooden pill-box with circular pieces of lint or linen, till it is above the level of the rim, keeping the rim clear. When antidyne is wanted, pour some of the liq. ammon. upon the lint or linen, so as to saturate the folds. The box is then to be instantly inverted on the part, and held on with firm but gentle pressure. It first feels like a piece of ice—in a minute or less, a sense of heat and tingling is experienced—then a burning heat—and in from two to five minutes a blister is raised. In fine, every physiological effect described by Dr. Granville is produced by this preparation. The lid is then to be pressed upon the box, and kept till another application is necessary, when some fresh liquor ammonia must be poured over the lint.

Now we think that this mode of application is infinitely preferable to that recommended by Dr. Granville—viz. wetting a pocket handkerchief, or several folds of linen, by which a great quantity of an expensive article is wasted, and the evaporation is not half so effectually prevented by the napkin as by the edge of the box, which leaves a dent in the skin like the rim of a cupping-glass, thus enabling any person to apply the ammonia close to the eye, if desirable, without the least risk of injury to that organ. This mode also prevents the great diffusion of ammoniacal gas through the room, which is very pungent and unpleasant to weak lungs or weak eyes. The only objection to this mode is the size of the box, which is too small, where a considerable surface is to be vesicated or counter-irritated. A wooden box, however, of any size, may be easily constructed;—or, the same box may be re-applied, with almost the celerity of a cupping-glass, and thus vesication or any degree of counter-irritation produced to any extent. The liq. ammonia should be kept in a very strong bottle well stopped, and in a wooden case. The bottle should not be more than half filled. We think that no medical man should be without this small but potent remedy, to be applied in cases of emergency, and where violent pain or spasm is to be quelled with celerity. We do not, indeed, believe that the remedy in question will ever prove extensively beneficial in chronic and painful maladies, where blisters or antimonial counter-irritants are now employed. The purulent discharge procured by lytta and antimony, or by setons, will always be more powerful derivatives than the caustic ammonia. But the latter will prove extremely useful in urgent spasms, or deep-seated inflammation of vital organs, where rapidity of agency is of the utmost importance.

In this brief analysis we have endeavoured to be useful to our readers rather than censorious or eulogistic towards the author. These latter propensities and practices we leave to our neighbours—and much good may they get by them. Dr. Granville, however, is entitled to thanks for the introduction of this powerful remedy. We now very frequently employ it.

---

